# Lactate/Albumin Ratio as a Predictor of In-Hospital Mortality in Septic Patients Presenting to the Emergency Department

**DOI:** 10.3389/fmed.2020.550182

**Published:** 2020-09-22

**Authors:** Ralphe Bou Chebl, Sarah Jamali, Mohamad Sabra, Rawan Safa, Iskandar Berbari, Ali Shami, Maha Makki, Hani Tamim, Gilbert Abou Dagher

**Affiliations:** ^1^Department of Emergency Medicine, American University of Beirut Medical Center, Beirut, Lebanon; ^2^Clinical Research Institute, American University of Beirut Medical Center, Beirut, Lebanon

**Keywords:** lactate/albumin ratio, lactate, albumin, mortality, sepsis, septic shock

## Abstract

**Background:** The aim of this study is to evaluate the prognostic value of the Lactate to Albumin (L/A) ratio compared to that of lactate only in predicting morbidity and mortality in sepsis patients.

**Methods:** This was a single-center retrospective cohort study. All adult patients above the age of 18 with a diagnosis of sepsis who presented between January 1, 2014 and June 30, 2019 were included. The primary outcome was in-hospital mortality.

**Results:** A total of 1,381 patients were included, 44% were female. Overall in-hospital mortality was 58.4% with the mortalities of sepsis and septic shock being 45.8 and 67%, respectively. 55.5% of patients were admitted to the intensive care unit. The area under the curve value for lactate was 0.61 (95% CI 0.57–0.65, *p* < 0.001) and for the L/A ratio was 0.67 (95% CI 0.63–0.70, *p* < 0.001). The cutoff generated was 1.22 (sensitivity 59%, specificity 62%) for the L/A ratio in all septic patients and 1.47 (sensitivity 60%, specificity 67%) in patients with septic shock. The L/A ratio was a predictor of in-hospital mortality (OR 1.53, CI 1.32–1.78, *p* < 0.001).

**Conclusion:** The L/A ratio has better prognostic performance than initial serum lactate for in-hospital mortality in adult septic patients.

## Learning Points

- This study aims to evaluate the prognostic value of the L/A ratio in predicting in-hospital mortality in septic patients compared to initial serum lactate. A total of 1,381 patients were included, 44% were female and their mean age was 71.2 years old.
- The L/A ratio was an independent predictor of in-hospital mortality (OR 1.53, CI 1.32–1.78, *p* < 0.001) in adult septic patients presenting to the emergency department.
- The optimal cutoff for the L/A ratio was found to be 1.22 (sensitivity 59%, specificity 62%) in septic patients and 1.47 (sensitivity 60%, specificity 67%) in patients with septic shock.
- The L/A ratio has better prognostic performance than initial serum lactate for predicting in-hospital mortality in adult patients with sepsis presenting to the emergency department.

## Introduction

### Background

Sepsis remains a major burden worldwide, with a global estimate of 31.5 million cases and 5.3 million deaths per year (1). It is responsible for ~35% of all hospital deaths, with overall mortality rates ranging between 20 and 30% (2, 3). The most critical parameters in sepsis management have been shown to be early recognition and timely broad-spectrum antibiotic administration (4–7). Management delays have been associated with increased mortality and morbidity (8, 9).

### Importance

Rapid recognition of high-risk patients remains a challenge, and multiple attempts are being made to identify readily available and cost-effective biomarkers to prognosticate and risk-stratify septic patients. An abundance of literature supports a strong independent association between serum lactate, a surrogate of tissue perfusion, and mortality among critically ill patients (10, 11). The current recommendation is to obtain a serum lactate level within the first hour of presentation in all patients with suspected sepsis, and to repeat the measurement within 2–4 h if the initial lactate is more than 2 mmol/L (12). However, lactic acidosis has been described in patients on metformin and albuterol (13), patients with diabetic ketoacidosis (14), malignancies (15), intoxication (16), hepatic or renal dysfunction (17, 18), and lastly, as a consequence of receiving epinephrine (13). In these cases, it is challenging to rely solely on lactate levels for prognostication. One emerging sepsis biomarker is the lactate to albumin (L/A) ratio. The addition of albumin and consideration of nutritional status in septic patients may address a weakness that exists in current major scoring systems such as the SOFA score (19). There is evidence that serum albumin correlates with morbidity and mortality in patients with critical illness (20). Given the limitations of lactate and the need for a surrogate marker of disease severity, a growing body of literature has found this ratio to be predictive of mortality and multiple organ failure in critically-ill patients with sepsis (7, 21, 22). Although promising, there is still a paucity of data and the L/A ratio requires further validation before it can be integrated into clinical practice.

### Goals of This Investigation

This study aimed to evaluate the prognostic value of the L/A ratio compared to lactate for predicting sepsis-related mortality in patients presenting to the emergency department (ED) of a tertiary medical center.

## Methods

### Study Design and Setting

This was a retrospective cohort study conducted in the academic emergency department (ED) of a tertiary care center between January 1, 2014 and June 30, 2019. Sepsis was defined according to the Third International Consensus Definitions for Sepsis and Septic Shock (Sepsis-3) guidelines as the presence of an infection with signs of organ dysfunction, which are represented by a Sequential [Sepsis-related] Organ Failure Assessment (SOFA) score of two points or greater (7). Septic shock was defined as a vasopressor requirement to maintain a mean arterial pressure of 65 mm Hg or greater, and a serum lactate level >2 mmol/L (>18 mg/dL) in the absence of hypovolemia (7). Institutional Review Board (IRB) approval was obtained (BIO-2018-0106), and due to the retrospective nature of the study informed consent was waived.

### Selection of Participants

Patients were identified through the hospital's electronic medical record system through their ICD-9 codes (International Statistical Classification of Diseases, Ninth Revision and Related Health Problems). All patient identifiers were removed during the data extraction process. All adult patients above the age of 18 with an ICD-9 diagnosis of sepsis (995.91) and septic shock (785.52) were included. Patients who met the criteria of sepsis-3 were included in the study. Exclusion criteria included patients identified as septic patients but did not meet sepsis-3 criteria, patients who did not have an albumin level, as well as patients admitted to the hospital and developed sepsis as a secondary diagnosis during their hospital stay.

### Data Collection

Data was extracted from patient's electronic medical record, anonymized and collected on a web-based secure report form. Prior to the data collection, a standardized protocol was established by the principal investigator for the data extraction process. Several meetings were held between the PI and the research team to standardize the data extraction method. Variables collected included patient characteristics, vital signs upon presentation to the ED, laboratory results, disposition, length of stay, mortality outcome, and interventions administered including antibiotics, mechanical ventilation, vasopressor and steroid use. Lactate levels were measured at ED presentation as per the latest sepsis-3 guidelines (7). Given that albumin's half-life is ~25 days, an albumin level was included in the study if it was measured in the ED or during the hospital admission (19, 23). The primary outcome was in-hospital mortality.

### Statistical Analysis

Statistical analysis was performed using IBM SPSS Statistics for Windows, version 24 (IBM Corp., Armonk, N.Y., USA) and Stata version 15 (College Station, TX, USA: StataCorp). Categorical variables are presented as frequency with percentages and continuous variables are presented as mean ± standard deviation. The receiver operative characteristic (ROC) curve was generated for both lactate and the L/A ratio. Patients were stratified into two groups: survivors and non-survivors. Youden's index was used to calculate the optimal cutoff values that predict hospital-mortality. Subgroup analyses were also done to look at the area under the ROC curves of both lactate and L/A ratios in septic shock patients, in patients with different lactate and albumin levels, and in patients with renal or hepatic dysfunction. Tests of linearity of both lactate and lactate/albumin ratio with the outcome were done as well as tests of interaction and colinearity between lactate and lactate/albumin ratio. We also performed a multivariable logistic regression to adjust for potential confounders in the association between the L/A ratio and in-hospital mortality. All variables with statistical significance and variables with clinical significance were included in the analysis. The variables included were age, gender, comorbidities of hepatic dysfunction, renal dysfunction, malignancy; coronary artery disease, dyslipidemia, atrial fibrillation, chronic kidney disease, end stage renal disease, vital signs SBP and DBP, laboratory values of hematocrit, BUN, creatinine, bicarbonate, calcium, phosphate, interventions of intubation, steroid administration, and vasopressor administration including the type of vasopressors administered (norepinephrine, dopamine, epinephrine, dobutamine).

## Results

### Patient Characteristics

A total of 3,932 septic patients were identified during the study period. Two thousand one hundred and fifty seven patients were excluded because there was no albumin drawn on them. Another 304 patients were excluded because they did not meet the sepsis-3 criteria. Finally, 90 patients were excluded because they were admitted for a non-infectious cause and developed sepsis during their hospital stay (admitted for stroke and orthopedic surgery). A total of 1,381 patients were included in this study, and baseline characteristics are presented in Table 1. Of these patients, 44% were female, 16.6% were smokers and their mean age was 71.2 ± 15.7 years old. There were a total of 806 (59.4%) patients with septic shock. The most common patient comorbidities were hypertension (64.2%), diabetes mellitus (37.4%), malignancy (37.3%) and coronary artery disease (33.1%). ED stay was on average 17 ± 22.5 h for the entire population, 15.1 ± 19.1 h in the survivor group and 19.7 ± 26.4 h in the non-survivor group (*p* < 0.001). Total hospital stay averaged 373.5 ± 512.1 h for the entire population, 300.5 ± 399.6 h for the survivor group and 475.9 ± 623.3 h in the non-survivor group (*p* < 0.001). Overall in-hospital mortality was 58.4% with the mortalities of sepsis and septic shock being 67 and 45.8%, respectively. More than half of the patients were admitted to the intensive care unit (ICU) (55.5%), and the non-survivor groups having a higher rates of ICU admission (72 vs. 43.8% *p* < 0.001).

**Table 1:** Baseline patient characteristics.

**Variable**	**Overall (*N* = 1381)**	**Survivors (*N* = 575)**	**Non-survivors (*N* = 806)**	***p***
	***n*****, %**			
Sex (female) Septic shock Sepsis	613 (44.4) 820 (59.4) 561 (40.6)	361 (44.8) 271 (33) 304(54.2)	252 (43.8) 549 (67) 257 (45.8)	0.723 <0.001 <0.001
Smoker	175 (16.6)	102 (16.5)	73 (16.8)	0.887
**Comorbidities**				
Hypertension	887 (64.2)	520 (64.5)	367 (63.8)	0.792
Diabetes mellitus	516 (37.4)	290 (36.0)	226 (39.3)	0.208
Malignancy	515 (37.3)	276 (34.2)	239 (41.6)	0.006
Coronary artery disease	457 (33.1)	245 (30.4)	212 (36.9)	0.011
Dyslipidemia	426 (30.9)	259 (32.1)	167 (29.1)	0.236
Diastolic HF	390 (30.2)	209 (27.5)	181 (34.0)	0.013
Atrial fibrillation	298 (21.6)	156 (19.4)	142 (24.7)	0.018
Chronic kidney disease	239 (17.7)	125 (15.8)	114 (20.3)	0.032
Systolic HF	208 (15.9)	89 (11.6)	119 (22)	<0.001
COPD	151 (10.9)	83 (10.3)	68 (11.8)	0.364
Cerebrovascular disease	88 (6.4)	45 (5.6)	43 (7.5)	0.154
End stage renal disease	83 (6.0)	38 (4.7)	45 (7.8)	0.016
Peripheral vascular disease	73 (5.3)	41 (5.1)	32 (5.6)	0.695
ICU admission	764 (55.5)	352 (43.8)	412 (72)	<0.001
	**Mean** **±** **SD**			
Age (years)	71.19 ± 15.73	70.52 ± 15.88	72.12 ± 15.48	0.063
**Vital signs at presentation**				
SBP (mm Hg)	115.09 ± 28.83	117.76 ± 28.63	111.34 ± 28.71	<0.001
DBP (mm Hg)	62.95 ± 18.10	63.52 ± 17.38	62.14 ± 19.04	0.163
HR (per minute)	102.54 ± 25.32	103.48 ± 24.66	101.21 ± 26.19	0.105
Oxygen saturation (%)	93.59 ± 7.91	94.56 ± 6.89	92.23 ± 8.99	<0.001
Temperature (°C)	37.36 ± 1.17	37.58 ± 1.17	37.04 ± 1.08	<0.001
Respiratory rate (per minute)	22.60 ± 6.41	21.82 ± 5.87	23.71 ± 6.95	<0.001
**Length of stay**				
ED (hours)	16.99 ± 22.51	15.09 ± 19.06	19.66 ± 26.41	<0.001
Total hospital (hours)	373.53 ± 512.07	300.46 ± 399.60	475.95 ± 623.27	<0.001

*°C, degrees Celsius; HF, heart failure; COPD, chronic obstructive pulmonary disease; SBP, systolic blood pressure; DBP, diastolic blood pressure; HR, heart rate; mmHg, millimeter of mercury; ED, emergency department; SD, standard deviation*.

### Laboratory and Vital Sign Results

Data on laboratory results is presented in Table 2. With regards to vital signs at presentation, the non-survivors showed a lower systolic blood pressure, a lower oxygen saturation, a lower temperature, and a higher respiratory rate compared to non-survivors (*p* < 0.001, all). The mean L/A ratio for all septic patients was 1.52 ± 1.37. The non-survivor group showed higher lactate levels (4.50 vs. 3.19 mmol/L), lower albumin (2.56 vs. 2.95), and a higher L/A ratio (1.93 vs. 1.20) than the survival subgroup (*p* < 0.001, all). The median time till albumin measurement was 6.5 h (IQR of 4.55). Furthermore, the non-survivor subgroup had higher rates of intubation at 24 and 48 h, steroid use and vasopressor use compared to survivors (*p* < 0.001, all).

**Table 2:** Patient laboratory results and interventions performed.

**Variable**	**Overall (*N* = 1381)**	**Survivors (*N* = 575) Mean ± SD**	**Non-survivors (*N* = 806)**	***p***
**Laboratory results**				
Lactate at presentation mmol/L	3.72 ± 3.03	3.19 ± 2.49	4.50 ± 3.53	<0.001
Albumin at presentation g/dL	2.77 ± 0.98	2.95 ± 0.66	2.56 ± 0.64	<0.001
Lactate/albumin ratio	1.52 ± 1.37	1.20 ± 1.03	1.93 ± 1.63	<0.001
Procalcitonin ng/ml	12.43 ± 26.29	12.60 ± 26.20	12.27 ± 26.42	0.884
Glucose mg/dL	158.33 ± 106.70	156.21 ± 107.57	161.35 ± 105.52	0.459
Hemoglobin g/dL	11.02 ± 2.22	11.17 ± 2.18	10.82 ± 2.25	0.003
Hematocrit %	33.24 ± 6.87	33.60 ± 6.75	32.74 ± 7.01	0.023
BUN mg/dl	39.37 ± 31.49	33.15 ± 26.19	48.11 ± 35.94	<0.001
Creatinine mg/dl	1.88 ± 1.80	1.69 ± 1.64	2.14 ± 1.96	<0.001
Baseline creatinine in patients with CKD mg/dL	1.95 ± 0.77	1.84 ± 0.65	2.08 ± 0.87	0.039
Sodium mmol/L	135.42 ± 6.53	135.46 ± 5.74	135.36 ± 7.50	0.793
Absolute neutrophil count /cu.mm	10594.95 ± 8139.66	10574.49 ± 7746.61	10623.76 ± 8669.79	0.912
Lymphocyte count %	13.00 ± 15.31	12.36 ± 14.76	13.91 ± 16.02	0.065
WBC /cu.mm	13237.84 ± 10013.68	13031.59 ± 9449.40	13528.25 ± 10759.99	0.365
Bicarbonate mmol/L	21.03 ± 5.99	21.54 ± 5.90	20.33 ± 6.05	<0.001
Chloride mmol/L	97.09 ± 8.25	97.29 ± 7.50	96.82 ± 9.19	0.318
Bilirubin total mg/dL	1.64 ± 3.56	1.44 ± 3.11	1.94 ± 4.13	0.088
Troponin ng/mL	0.11 ± 0.19	0.087 ± 0.17	0.133 ± 0.20	0.015
Potassium mmol/L	4.79 ± 6/04	4.90 ± 7.88	4.64 ± 0.98	0.434
Magnesium mg/dL	2.20 ± 5.42	2.22 ± 6.29	2.18 ± 3.90	0.896
Calcium mg/dL	8.68 ± 2.68	8.81 ± 3.38	8.51 ± 1.12	0.040
Phosphate mg/dL	3.84 ± 3.21	3.46 ± 3.77	4.36 ± 2.09	<0.001
pH (Arterial)	7.35 ± 0.11	7.36 ± 0.10	7.34 ± 0.12	0.017
PaCO_2_ mmHg	35.90 ± 15.42	35.74 ± 16.47	36.06 ± 14.39	0.818
PT seconds	20.76 ± 15.91	19.13 ± 15.03	22.45 ± 16.62	0.005
PTT seconds	36.17 ± 23.47	34.73 ± 23.66	37.66 ± 23.21	0.096
INR	1.85 ± 1.84	1.71 ± 1.66	1.99 ± 240	0.054
**Interventions**				
IV fluids in first 6 h (L)	2.26 ± 1.84	2.25 ± 1.80	2.28 ± 1.90	0.724
IV fluids in first 24 h (L)	3.39 ± 2.23	3.35 ± 2.19	3.46 ± 2.28	0.388
	***n*****, %**			
Vasopressor use	431 (31.3)	205 (25.5)	255 (39.6)	<0.001
Norepinephrine	412 (29.8)	199 (24.7)	213 (37.0)	<0.001
Dopamine	35 (2.5)	8 (1.0)	27 (4.7)	<0.001
Epinephrine	46 (3.3)	17 (2.1)	29 (5.0)	0.003
Dobutamine	18 (1.3)	5 (0.6)	13 (2.3)	0.008
Steroid use	273 (19.9)	135 (16.8)	138 (24.2)	0.001
Q-sofa, >2	1077 (78)	416 (72.3)	661 (82)	<0.001
Intubation in first 24 h	262 (19.1)	83 (10.3)	179 (31.4)	<0.001
Intubation in first 48 h	183 (13.3)	61 (7.6)	122 (21.4)	<0.001

*/cu.mm, per cubic millimeter; CKD, chronic kidney disease; g/dL, grams per deciliter; g/L, grams per liter; INR, International Normalized Ratio; mg/dL, milligrams per deciliter; mmHg, millimeter of mercury; mmol/L, millimoles per liter; ng/ml, nanograms per milliliter; PaCO_2_, partial pressure of carbon dioxide; PT, Prothrombin Time; PTT, Partial Thromboplastin Time; SD, standard deviation; WBC, white blood cells; IV, intravenous; L, liter*.

### Infection Details

Information on the details of the infections can be found in Table 3. The median time until antibiotic administration was 2.22 h (interquartile range 3.69) and the most common infection sites were respiratory (43.0%) followed by the urinary tract (31.4%). *Escherichia coli* was the most commonly implicated bacteria in blood (17.8%), urine (18.6%), sputum (2.5%), and wound cultures (2.2%).

**Table 3:** Infection characteristics.

**Variable**	**Overall (*N* = 1381)**	**Survivors (*N* = 575) Median ± IQR**	**Non-survivors (*N* = 806)**	***p***
Time to antibiotic (hours)	2.22 ± 3.69	2.14 ± 3.29	2.40 ± 4.105	0.157
	***n*****, %**	
Appropriate choice of antibiotic	1112 (93.6)	674 (94.5)	438 (92.2)	0.109
**Infection site**				
Respiratory	584 (43.0)	289 (36.3)	295 (52.6)	<0.001
Urinary tract	429 (31.4)	279 (34.8)	150 (26.6)	0.001
Intravascular indwelling device	280 (20.6)	198 (24.8)	82 (14.6)	<0.001
Gastrointestinal	227 (16.6)	133 (16.6)	94 (16.7)	0.984
Skin	64 (4.7)	43 (5.4)	21 (3.7)	0.160
Heart	38 (2.8)	20 (2.5)	18 (3.2)	0.447
Gallbladder	33 (2.4)	23 (2.9)	10 (1.8)	0.192
Surgical site	25 (1.8)	10 (1.3)	15 (2.7)	0.056
Bone	5 (0.4)	5 (0.6)	0 (0.0)	0.081
Other or unknown	38 (2.8)	25 (3.1)	13 (2.3)	0.361
Bacteria identified in blood	613 (44.6)	460 (57.3)	153 (26.8)	<0.001
*E. coli*	246 (17.8)	197 (24.4)	49 (8.5)	<0.001
*Staphylococcus* Coagulase negative	150 (10.9)	109 (13.5)	41 (7.1)	<0.001
*Streptococcus*	52 (3.8)	38 (4.7)	14 (2.4)	0.028
*Staphylococcus aureus*	22 (1.6)	17 (2.1)	5 (0.9)	0.070
Other	206 (14.9)	139 (17.2)	67 (11.7)	0.004
Bacteria identified in urine	392 (28.5)	245 (30.5)	147 (25.7)	0.051
*E. coli*	257 (18.6)	171 (21.2)	86 (15.0)	0.003
*Klebsiella pneumoniae*	61 (4.4)	38 (4.7)	23 (4.0)	0.524
*Acinetobacter baumannii*	14 (1.0)	4 (0.5)	10 (1.7)	0.023
Other	120 (8.7)	62 (7.7)	58 (10.1)	0.119
Bacteria identified in sputum	156 (11.5)	52 (6.6)	104 (18.2)	<0.001
*E. coli*	35 (2.5)	10 (1.2)	25 (4.3)	<0.001
*Pseudomonas aeruginosa*	29 (2.1)	13 (1.6)	16 (2.8)	0.135
*A. baumannii*	28 (2.0)	5 (0.6)	23 (4.0)	<0.001
Other	93 (6.7)	31 (3.8)	62 (10.8)	<0.001
Bacteria identified in wound	86 (6.3)	41 (5.2)	45 (7.9)	0.044
*E. coli*	31 (2.2)	13 (1.6)	18 (3.1)	0.061
*Enterococcus*	15 (1.1)	4 (0.5)	11 (1.9)	0.012
*Staphylococcus* Coagulase negative	12 (0.9)	6 (0.7)	6 (1.0)	0.570
*Pseudomonas aeruginosa*	11 (0.8)	6 (0.6)	6 (1.0)	0.541
*S. aureus*	9 (0.7)	3 (0.4)	6 (1.0)	0.176
*Streptococcus*	7 (0.5)	6 (0.7)	1 (0.2)	0.250
Other	49 (3.5)	23 (2.9)	26 (4.5)	0.099

*IQR, interquartile range*.

### Area Under the Receiver Operating Curve

**Figure 1:**
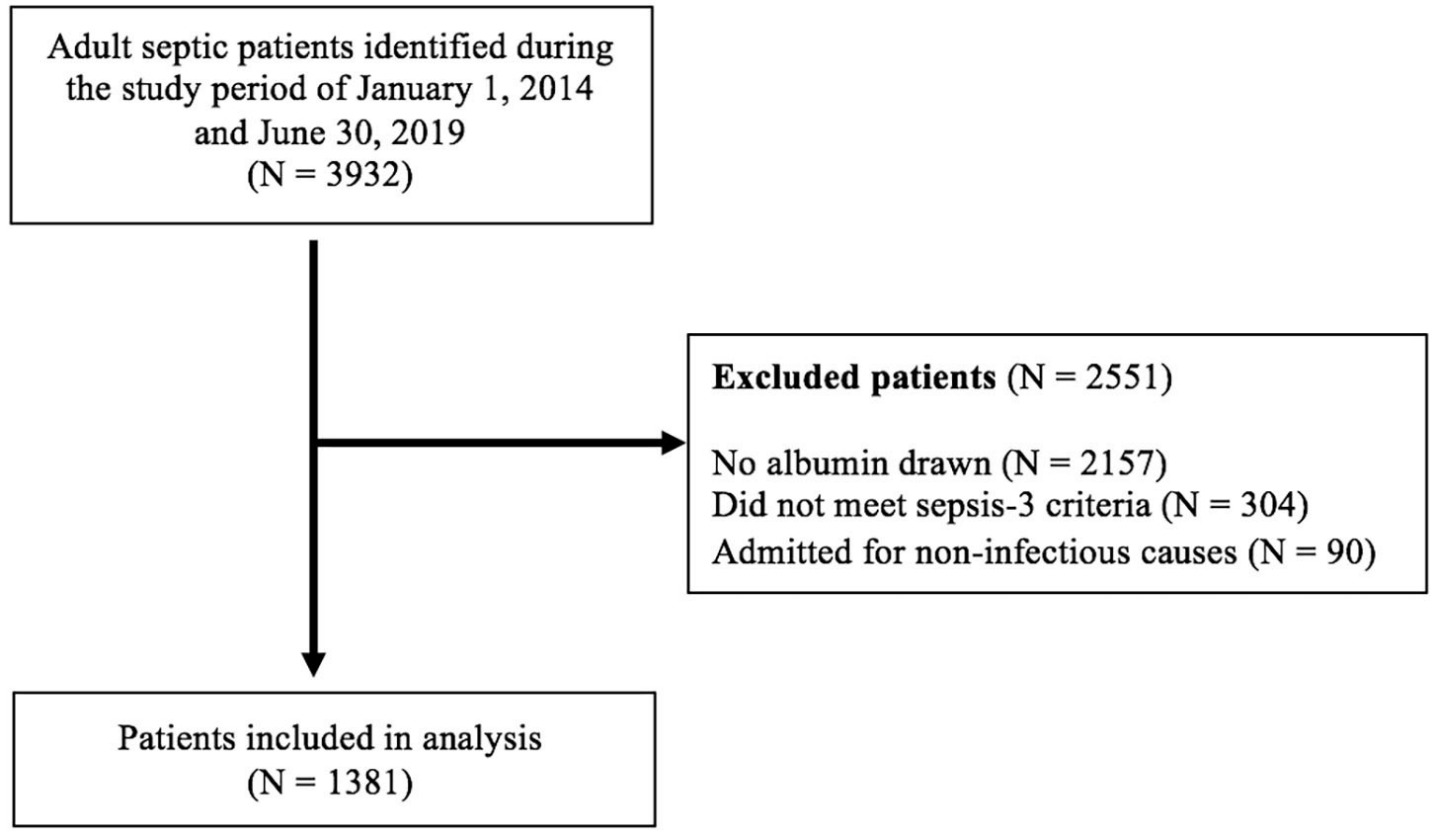
Flow diagram of patient selection.

**Figure 2:**
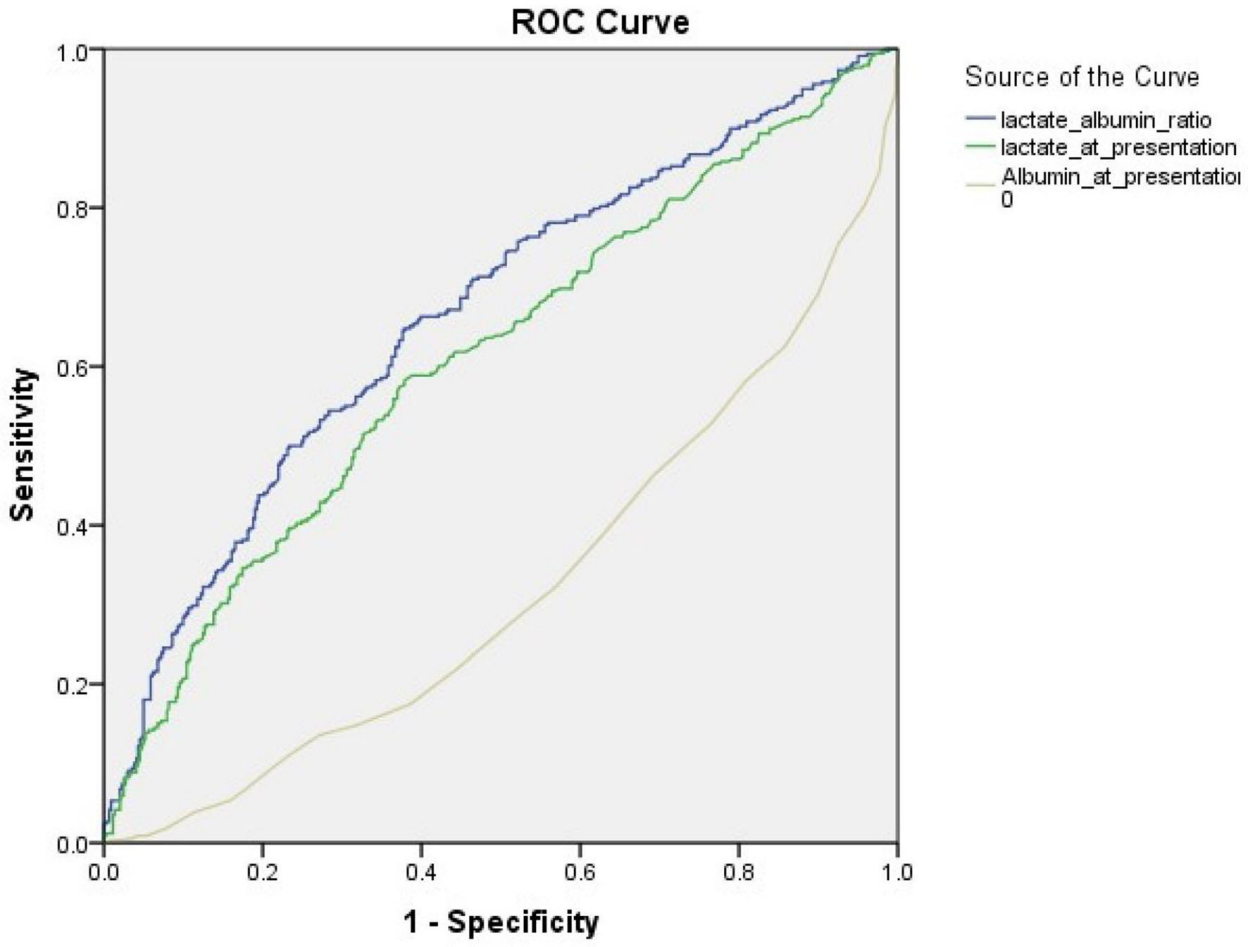
Receiver operating characteristic curves for in-hospital mortality among all septic patients. The area under the receive operating curve (AUC) for in-hospital mortality among all septic patients. The AUC for lactate is 0.61 (95% CI 0.57–0.65) and that for the lactate/albumin ratio is 0.67 (95% CI 0.63–0.70, *p* < 0.001).

**Figure 3:**
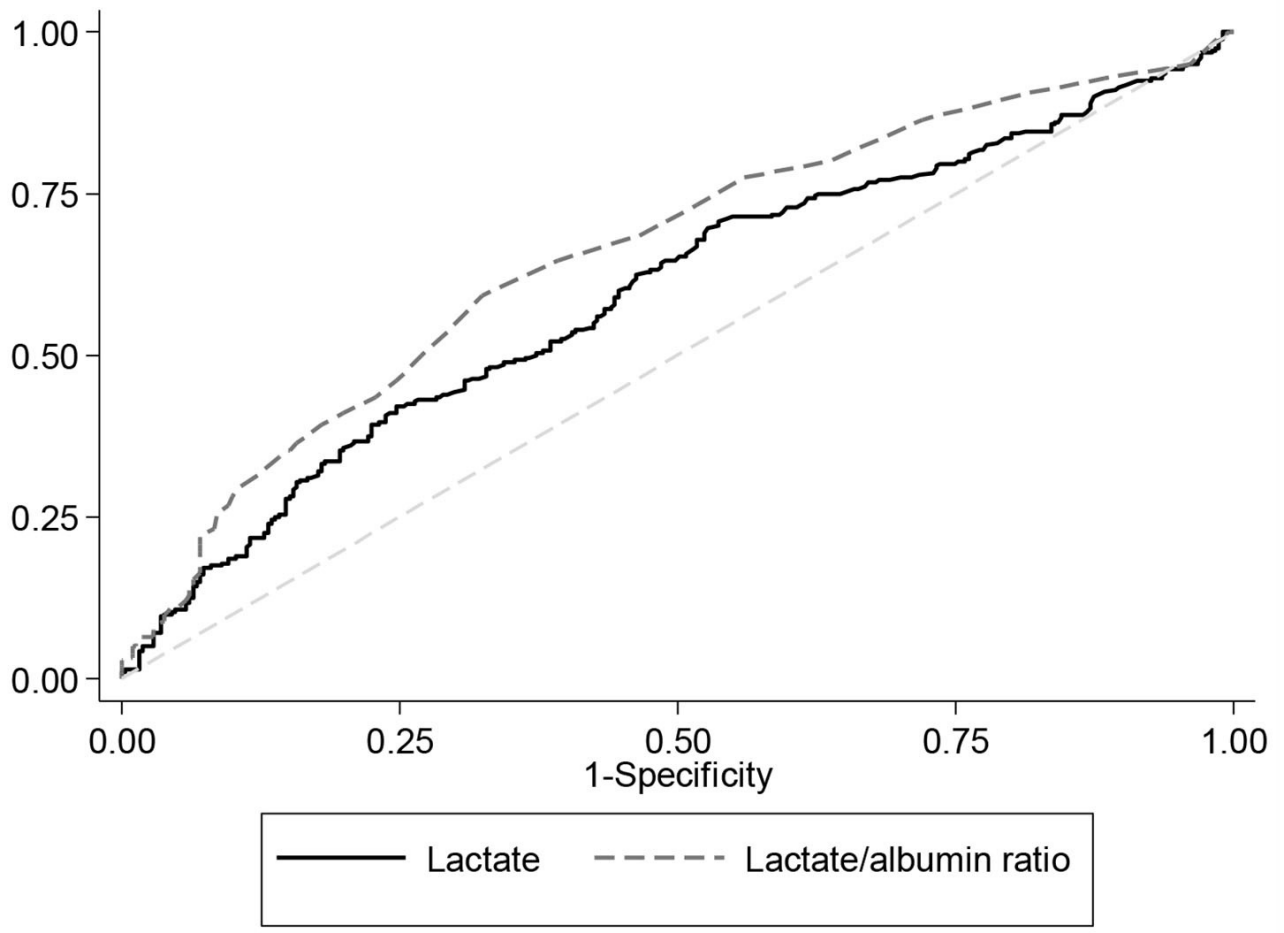
Receiver operating characteristic curves for in-hospital mortality among patients in septic shock. The area under the receive operating curve (AUC) for in-hospital mortality among patients with septic shock. The AUC for lactate is 0.59 (95% CI 0.55–0.64) and that for the lactate/albumin ratio is 0.66 (95% CI 0.61–0.70, *p* < 0.001).

Figure 1 illustrates the area under the curve (AUC) values for lactate only compared to that of the L/A ratio for predicting in-hospital mortality. Table 4 presents the AUC values for lactate and the L/A ratio for predicting in-hospital mortality across different patient subgroups. The AUC value of the L/A ratio in all patients was 0.67 (95% CI 0.63–0.70, *p* < 0.001) and is higher than that of lactate alone 0.61 (95% CI 0.57–0.65, *p* < 0.001) as well as that of albumin alone of 0.34 (95% CI 0.29–0.36, *p* < 0.001) (Figure 2). Among patients with septic shock, the AUC for the L/A ratio was 0.66 (95% CI 0.61–0.70, *p* < 0.001) and was higher than the AUC of lactate only (0.59, 95% CI 0.55–0.64, *p* < 0.001) (Figure 3). The AUC of the L/A ratio was significantly higher than that of lactate alone regardless of the lactate level (lactate <2 mmol/L: 0.63, 95% CI 0.55–0.71 vs. 0.50, 95% CI 0.43–0.58, *p* = 0.001, lactate ≥2 mmol/L: 0.67, 95% CI 0.63–0.72 vs. 0.61, 95% CI 0.56–0.65, *p* < 0.001). A significantly higher prognostic value of the L/A ratio was observed in the renal dysfunction subgroup (0.62, 95% CI 0.54–0.71 vs. 0.58, 95% CI 90.50–0.67, *p* = 0.009). Youden's index was employed to calculate the optimal L/A ratio cutoff threshold and found it to be 1.22 for all septic patients (positive predictive value 57%, negative predictive value 70%) and 1.47 for patients with septic shock (positive predictive value 62%, negative predictive value 65%).

**Table 4:** Area under the curve (AUC) and lactate/albumin ratio cutoff thresholds for in-hospital mortality among different patient subgroups.

	**AUC for in-hospital mortality (95% CI)**			**Lactate/albumin ratio cut-off threshold**				
	**Lactate**	**L/A ratio**	***p***	**Cut-off threshold**	**Sensitivity**	**Specificity**	**PPV**	**NPV**
Overall	0.61 (0.57–0.65)	0.67 (0.63–0.70)	<0.001	1.22	0.59	0.62	0.57	0.70
Septic shock	0.59 (0.55–0.64)	0.66 (0.61–0.70)	<0.001	1.47	0.60	0.67	0.62	0.65
**Lactate levels**								
Lactate <2 mmol/L	0.50 (0.43–0.58)	0.63 (0.55–0.71)	0.001	0.54	0.63	0.58	0.82	0.71
Lactate ≥2 mmol/L	0.61 (0.56–0.65)	0.67 (0.63–0.72)	<0.001	1.44	0.65	0.65	0.62	0.67
**Albumin levels**								
Albumin <3 g/dL	0.61 (0.56–0.66)	0.65 (0.60–0.69)	<0.001	1.44	0.54	0.69	0.66	0.58
Albumin ≥3 g/dL	0.58 (0.50–0.66)	0.59 (0.51–0.67)	0.42	1.47	0.32	0.87	0.48	0.77
**Patient subgroups**								
Renal dysfunction	0.58 (0.50–0.67)	0.62 (0.54–0.71)	0.009	1.51	0.36	0.87	0.71	0.59
Hepatic dysfunction	0.55 (0.29–0.82)	0.60 (0.33–0.86)	0.27	1.41	0.64	0.71	0.82	0.50
**Infection site**								
Respiratory	0.62 (0.56–0.68)	0.66 (0.61–0.72)	<0.001	1.39	0.51	0.79	0.73	0.59
Urinary tract	0.56 (0.48–0.63)	0.64 (0.57–0.71)	<0.001	0.85	0.69	0.54	0.50	0.73
Intravascular indwelling device	0.61 (0.52–0.71)	0.69 (0.60–0.78)	<0.001	1.44	0.64	0.71	0.51	0.81
Gastrointestinal	0.71 (0.62–0.81)	0.75 (0.66–0.85)	<0.001	1.43	0.63	0.83	0.70	0.78
Skin	0.62 (0.43–0.82)	0.69 (0.51–0.88)	0.06	1.08	0.93	0.58	0.62	0.92
Heart	0.78 (0.54–1.00)	0.89 (0.72–1.00)	0.01	0.83	0.78	1.00	1.00	0.78
Gallbladder	0.49 (0.19–0.78)	0.46 (0.14–0.77)	0.76	2.40	0.17	1.00	1.00	0.75
Surgical site	0.52 (0.30–0.87)	0.58 (0.28–0.88)	0.56	1.64	0.64	0.71	0.78	0.56

*PPV = positive predictive value, NPV = negative predictive value*.

### Multivariate Logistic Regression

The results of the multivariable logistic regression can be found in Table 5. After adjusting for multiple confounding variables such as age, sex, comorbidities, laboratory results, and interventions, we found that every one unit increase in the L/A ratio, increases by 50% the odds of in-hospital mortality (OR 4.56, 95% CI 2.76–7.52, *p* < 0.001).

**Table 5:** Multivariable logistic regression for in-hospital mortality with all statistically and clinically significant variables.

	**OR**		**95% C.I**.	***P*-value**
		**Lower**	**Upper**	
Lactate/albumin ratio	4.56	2.76	7.52	0.000
Lactate	0.70	0.57	0.86	0.001
Interaction of lactate and lactate ratio	0.98	0.96	1.01	0.153
Age	1.00	0.99	1.01	0.983
Gender	1.07	0.75	1.52	0.706
Hepatic dysfunction	2.39	0.82	7.00	0.112
Cancer	1.62	1.10	2.37	0.014
Coronary artery disease	1.38	0.92	2.05	0.116
Dyslipidemia	1.00	0.67	1.48	0.995
Atrial fibrillation	1.35	0.89	2.05	0.163
Chronic kidney disease	0.96	0.60	1.55	0.876
SBP	1.00	1.00	1.01	0.306
DBP	1.01	0.99	1.02	0.362
End stage renal disease	4.24	1.59	11.31	0.004
Hematocrit	1.00	0.97	1.02	0.818
BUN	1.01	1.01	1.02	0.000
Creatinine	0.84	0.71	1.00	0.047
Bicarbonate	1.05	1.01	1.08	0.015
Calcium	0.79	0.71	0.89	0.000
Phosphate	1.24	1.12	1.37	0.000
Vasopressors in first 24 h	0.69	0.09	5.20	0.715
Vasopressors in first 24 h–Norepinephrine	1.20	0.16	9.04	0.859
Vasopressors in first 24 h–Dopamine	7.32	1.40	38.20	0.018
Vasopressors in first 24 h–Epinepherine	0.80	0.28	2.25	0.666
Dobutamine in first 24 h	1.88	0.38	9.39	0.441
Steroids in first 24 h	1.11	0.73	1.68	0.635
Intubation in first 24 h	2.00	1.11	3.59	0.021
Intubation in first 48 h	0.99	0.53	1.83	0.965

## Discussion

The results of this study have shown that the L/A ratio has better prognostic performance for predicting in-hospital mortality in septic patients. When looking at only the septic shock population, the L/A ratio also outperformed lactate alone (AUC 0.66 vs. 0.59). Improved prognostic accuracy was consistent across subgroups of lactate levels (lactate <2 mmol/L and lactate ≥2 mmol/L), hypoalbuminemia (albumin <3 g/dL) and renal dysfunction. Additionally, the L/A ratio was an independent predictor of mortality (OR 4.56, *p* < 0.001). Our study supports existing literature on the use of the L/A ratio as a prognostic marker in patients with sepsis with fair discriminative value. Although lactate is a well-studied prognostic biomarker, its interpretation is complex due to the pathophysiology that can lead to serum lactate elevations (13–18). Moreover, normal lactate levels may be falsely misinterpreted as a good prognosis in high-risk patients. As such, the incorporation of albumin as a reflection of nutritional status in the L/A ratio increases its use in prognostication of sepsis patients.

Several studies have shown that the L/A ratio has good discriminatory power when comparing it to lactate (24). Lichtenauer et al. (21) also evaluated adult ICU patients and had similar findings (*n* = 348) and found fair discriminative ability for in-hospital mortality with an AUC of 0.70 for the L/A ratio. Moustafa et al. (25) recently conducted a prospective study in pediatric patients admitted to the ICU with severe sepsis or septic shock (*n* = 119). They found that the L/A ratio at ED presentation correlates with mortality (AUC 0.681) and that it has better prognostic ability than lactate clearance at 6 and 24 h, especially in patients with hepatic dysfunction. It is worth noting that these studies are limited by their small sample size. The largest study was done by Shin et al. (22) who retrieved data from a multi-center registry of 10 EDs and, to the best of our knowledge, had the largest collective of patients analyzed for the relevance of the L/A ratio prior to this study (*n* = 946). Their AUC for the L/A ratio was 0.69 and was significantly higher than that of lactate (0.62) for predicting 28-day mortality, and these results are comparable with ours and reinforce the finding that the lactate to albumin ratio outperforms lactate alone as a prognostic marker in sepsis. Our study also looked at the prognostic role of L/A in specific subpopulation such as patients with hepatic or renal dysfunction. Given that lactate is primarily metabolized by the liver and to a lesser extent by the kidney, these patients often present to the Emergency Department with elevated lactate (26). Furthermore, because of impaired clearance, physicians are also faced with a persistently elevated lactate in these patients and often face the question of when to stop fluid resuscitation (26, 27). It is important to note that L/A ratio outperformed lactate alone in these subpopulation as well as patients with poor nutritional status (albumin <3g/dL), and physicians should probably consider relying on the ratio instead of lactate alone.

Furthermore, an interesting finding in our study is that the ratio outperformed lactate alone when we looked at different infection types. We found that the ratio had a better discriminatory power in the setting of genitourinary (GU), gastrointestinal and pulmonary infections. However, the difference was not found to be statistically significant for the surgical infections. One possible explanation for the improved discriminatory power could be that urinary and pulmonary infections are very common in elderly patients with poor nutritional status at baseline as opposed to patients with surgical infections (cholecystitis and appendicitis) who are usually younger, healthier patients.

One interesting finding in our study was the association hyperphosphatemia, hypercalcemia and elevated BUN with increased mortality. The ROC curves for phosphate, calcium and BUN were found to be 0.65 (95% CI 0.63–0.69, *p* < 0.0001), 0.42 (95% CI 0.39–0.46, *p* < 0.0001), and 0.63 (95% CI 0.61–0.68, *p* < 0.0001), respectively. While the ROC curve for calcium was lower than lactate and L/A's, the association between hyperphosphatemia and BUN with increased mortality is well-documented in the literature (28, 29). A study by Haider et al. (29) found that hyperphosphatemia was an independent predictor of 28-day mortality in critically ill patients, and this was probably due to the increased atherosclerosis and increased cardiovascular adverse events associated with hyperphosphatemia. In a similar fashion, Beier et al. (28) found that patients with blood urea nitrogen of >40 mg/dL had an odds ratio for mortality of 2.78 (95% confidence interval, 2.27–3.39; *p* < 0.0001) relative to patients with blood urea nitrogen of 10–20 mg/dL. It should be noted that are secondary outcomes and as such, they should be interpreted cautiously, until dedicated prospective studies evaluate their prognostic role.

The optimal cutoff value of the L/A ratio that discriminates survivors from non-survivors was 1.22 (PPV 57%, NPV 70%) for all septic patients and 1.47 (PPV 62%, NPV 65%) for patients with septic shock. Shin et al. (22) employed the Liu method and found an optimal cutoff value of 1.32 which is slightly higher than our findings. The optimal cutoff value of L/A ratio remains unknown and the authors recommend the need for a future study that is prospective in nature to help establish the optimal L/A ratio.

### Limitations

There were several limitations to this study. First, this study was retrospective in nature and thus has inherent limitations with regards to selection bias. The authors were aware of the potential biases held multiple meetings to ensure patients were correctly identified, minimize the patients who were missed due to improper ICD-9 classification, and standardize the data collection protocol. Second, the data was collected from a single center limiting the generalizability of these results. Third, we only included patients on whom an albumin was taken. Albumin levels are not routinely drawn on septic patients in the ED but are usually done to evaluate a patient's nutritional status. Having restricted our inclusion to patients with albumin levels might have introduced a selection bias for patients with a poor nutritional status. This selection bias might explain the high mortality seen in our study which is not representative of the sepsis-related mortality that is reported in the literature (4–6). Finally, we did not have enough information to compare the performance of the L/A ratio with validated ICU scoring systems such as APACHE II as well as other prognostic markers often used in sepsis such as procalcitonin and CRP. Finally, we do not have any information about the percentage of patients who worsened from sepsis to septic shock. As such, we cannot comment about the prognostic value of the lactate to albumin ratio in predicting the progression rate from sepsis to septic shock. Prospective studies with large populations and multiple centers would be an appropriate next step in the evaluation of the L/A ratio, and in determining the diagnostic sensitivity, specificity and positive and negative predictive value of the L/A ratio cutoff.

## Conclusion

In summary, the L/A ratio is a readily available parameter with consistently better prognostic performance than initial serum lactate for in-hospital mortality in adult patients with sepsis. We believe that the L/A ratio has additive value to the established risk stratification models.

## Data Availability Statement

The raw data supporting the conclusions of this article will be made available by the authors, without undue reservation.

## Ethics Statement

The studies involving human participants were reviewed and approved by the American University of Beirut Institutional Review Board (IRB) (BIO-2018-0106). Written informed consent for participation was not required for this study in accordance with the national legislation and the institutional requirements.

## Author Contributions

RB, GA, SJ, HT, and MM made substantial contributions to the conception and design of the study. RB, SJ, RS, IB, MS, AS, HT, and MM were responsible for acquisition of data, analysis and interpretation of data. RB, GA, IB, RS, and SJ were involved in drafting the manuscript. GA, MS, and AS were responsible for revising the manuscript critically for important intellectual content. RB took responsibility for the paper as a whole. All authors contributed to the article and approved the submitted version.

## Conflict of Interest

The authors declare that the research was conducted in the absence of any commercial or financial relationships that could be construed as a potential conflict of interest.

## Abbreviations

%: percentage
L/A ratio: lactate to albumin ratio
BUN: blood urea nitrogen
CI: Confidence interval
COPD: chronic obstructive pulmonary disease
HF: heart failure
CKD: chronic kidney disease
SBP: systolic blood pressure
DBP: diastolic blood pressure
ED: emergency department
mg/dL: milligram per deciliter
g/L: grams per liter
g/dL: grams per deciliter
HR: heart rate
ICD: International Statistical Classification of Diseases
ICU: intensive care unit
IV: intravenous
L: liter
mmHg: millimeter of mercury
mmol/L: millimoles per liter
ng/ml: nanogram per milliliter
PaCO_2_: partial pressure of carbon dioxide
PT: Prothrombin Time
PTT: Partial Thromboplastin Time
SD: Standard deviation
SOFA: Sequential [Sepsis-related] Organ Failure Assessment
FiO_2_: fraction of inspired oxygen
PaO_2_: partial pressure of oxygen in arterial blood
APACHE II: Acute Physiology and Chronic Health Evaluation II score
AUC: area under the curve
ROC: receiver operating curve
WBC: white blood cells
°C: degrees Celsius
IRB: institutional review board.
